# Fitness and mobility training in patients with Intensive Care Unit-acquired muscle weakness (FITonICU): study protocol for a randomised controlled trial

**DOI:** 10.1186/s13063-016-1687-4

**Published:** 2016-11-24

**Authors:** Jan Mehrholz, Simone Thomas, Jane H. Burridge, André Schmidt, Bettina Scheffler, Ralph Schellin, Stefan Rückriem, Daniel Meißner, Katja Mehrholz, Wolfgang Sauter, Ulf Bodechtel, Bernhard Elsner

**Affiliations:** 1 Wissenschaftliches Institut, Private Europäische Medizinische Akademie der Klinik Bavaria in Kreischa, An der Wolfsschlucht 1-2, Kreischa, 01731 Germany; 2 Department of Public Health, Medizinische Fakultät, Carl Gustav Carus, Technische Universität, Dresden, Germany; 3 Neurorehabilitation Research Group, Faculty of Health Sciences, University of Southampton, Southampton, UK; 4 Fach und Privatkrankenhaus, Klinik Bavaria in Kreischa, An der Wolfsschlucht 1-2, Kreischa, 01731 Germany

**Keywords:** Physical rehabilitation, ICU-acquired muscle weakness, Activities of daily living

## Abstract

**Background:**

Critical illness myopathy (CIM) and polyneuropathy (CIP) are a common complication of critical illness. Both cause intensive-care-unit-acquired (ICU-acquired) muscle weakness (ICUAW) which increases morbidity and delays rehabilitation and recovery of activities of daily living such as walking ability. Focused physical rehabilitation of people with ICUAW is, therefore, of great importance at both an individual and a societal level. A recent systematic Cochrane review found no randomised controlled trials (RCT), and thus no supporting evidence, for physical rehabilitation interventions for people with defined CIP and CIM to improve activities of daily living. Therefore, the aim of our study is to compare the effects of an additional physiotherapy programme with systematically augmented levels of mobilisation with additional in-bed cycling (as the parallel group) on walking and other activities of daily living.

**Methods/design:**

We will conduct a prospective, rater-masked RCT of people with ICUAW with a defined diagnosis of CIM and/or CIP in our post-acute hospital. We will randomly assign patients to one of two parallel groups in a 1:1 ratio and will use a concealed allocation. One intervention group will receive, in addition to standard ICU treatment, physiotherapy with systematically augmented levels of mobilisation (five times per week, over 2 weeks; 20 min each session; with a total of 10 additional sessions). The other intervention group will receive, in addition to standard ICU treatment, in-bed cycle sessions (same number, frequency and treatment time as the intervention group).

Standard ICU treatment includes sitting balance exercise, stretching, positioning, and sit-to-stand training, and transfer training to get out of bed, strengthening exercise (in and out of bed), and stepping and assistive standing exercises.

Primary efficacy endpoints will be walking ability (defined as a Functional Ambulation Category (FAC) level of ≥3) and the sum score of the Functional Status Score for the Intensive Care Unit (FSS-ICU) (range 0–22 points) assessed by a blinded tester immediately after 2 weeks of additional therapy.

Secondary outcomes will include assessment of sit-to-stand recovery, overall limb strength (Medical Research Council, MRC) and grip strength, the Physical Function for the Intensive Care Unit Test-Scored (PFIT-S), the EuroQol 5 Dimensions (EQ-5D) questionnaire and the Reintegration to Normal Living Index (RNL-Index) assessed by a blinded tester.

We will measure primary and secondary outcomes with blinded assessors at baseline, immediately after 2 weeks of additional therapy, and at 3 weeks and 6 months and 12 months after the end of the additional therapy intervention.

Based on our sample size calculation 108 patients will be recruited from our post-acute ICU in the next 3 to 4 years.

**Discussion:**

This will be the first RCT comparing the effects of two physical rehabilitation interventions for people with ICUAW due to defined CIP and/or CIM to improve walking and other activities of daily living. The results of this trial will provide robust evidence for physical rehabilitation of people with CIP and/or CIP who often require long-term care.

**Trial registration:**

We registered the study on 6 April 2016 before enrolling the first patient in the trial at the German Clinical Trials Register (www.germanctr.de) with the identifier DRKS00010269. This is the first version of the protocol (FITonICU study protocol).

**Electronic supplementary material:**

The online version of this article (doi:10.1186/s13063-016-1687-4) contains supplementary material, which is available to authorized users.

## Background

Critical illness myopathy (CIM) and polyneuropathy (CIP) are common results of critical illness that frequently occur together. Both cause so-called intensive-care-unit-acquired (ICU-acquired) muscle weakness (ICUAW). Such acquired muscle weakness is characterised by a profound weakness that is greater than normally expected from prolonged bed rest and, therefore, designated as clinically detected weakness in critically ill patients in whom there is no plausible aetiology other than critical illness [1–3]. The weakness of limb muscles significantly limits activities, such as sit-to stand, and assistance in transfers is often required [3–6]. This in turn increases morbidity and delays rehabilitation and recovery of walking [7–9]. Although full recovery has been reported in approximately 50% of people with ICUAW, improvement is related to the severity of the condition, e.g. people with severe weakness may take months to improve, or even remain severely affected [3, 10]. Focused physical rehabilitation of people with ICUAW is, therefore, of great importance. There is practical evidence that physical rehabilitation of patients can be implemented with few adverse effects [1, 11, 12]. Recently, appropriate assessments were developed and descriptions of suitable physical intervention strategies were described in the medical literature [1, 9, 11, 13–16]. Until now many of the described therapies investigated people in the acute stage of ICU treatment [17] with only a few trials considering patients who are so-called chronically critically ill [18] and in the post-acute phase or people in the post-ICU phase [18–20]. There is, therefore, still an urgent need for effective therapy and rehabilitation in the long-term after intensive care, e.g. to improve physical function [20].

### The need for a trial on rehabilitation interventions for people in the post-acute phase with ICUAW

As it seems clear that physical activity may be a promising intervention to address functional challenges for people with ICUAW due to a defined CIP and/or CIM, it is surprising that a recent systematic Cochrane review found no RCTs that examined the effects of physical rehabilitation interventions for people with ICUAW and defined CIP and/or CIM [21]. Several key questions need to be answered. Firstly, is one of two tailored, additional physical interventions more effective in helping people with ICUAW and defined CIP and/or CIM to improve activities of daily living and walking? Secondly, does the range of possible benefits include strength and quality of life, immediately after intervention and on follow-up? Thirdly, what is the optimal and effective physical intervention prescription for people in the post-acute ICU with ICUAW and defined CIP and/or CIM?

### Objective

The objective of the Fitness and mobility training in patients with Intensive Care Unit-acquired muscle weakness (FITonICU) study is, therefore, to describe the effects of an additional physiotherapy programme, with systematically augmented levels of mobilisation, compared with additional in-bed cycling on walking and other activities of daily living. The null hypothesis is that there is no statistically significant difference in the primary outcomes for the applied additional interventions.

We chose these comparators because both mobilisation and in-bed cycling are physical rehabilitation interventions that are commonly used clinically and seem feasible and suitable for these chronically critically ill patients in the post-acute ICU setting. Examples here include (additional) in-bed cycling often being used in critically ill patients on ICU [22] and also in the post-acute phase [18] and a physiotherapy programme with augmented levels of mobilisation is likely to be desirable as mobilisation is often used in the rehabilitation of chronically critically ill patients [3].

We will use a parallel-group randomised controlled trial (RCT) design to investigate the effects of these two different physical rehabilitation interventions for people with ICUAW due to defined CIP and/or CIM.

## Methods/design

### Study design

**Fig. 1:**
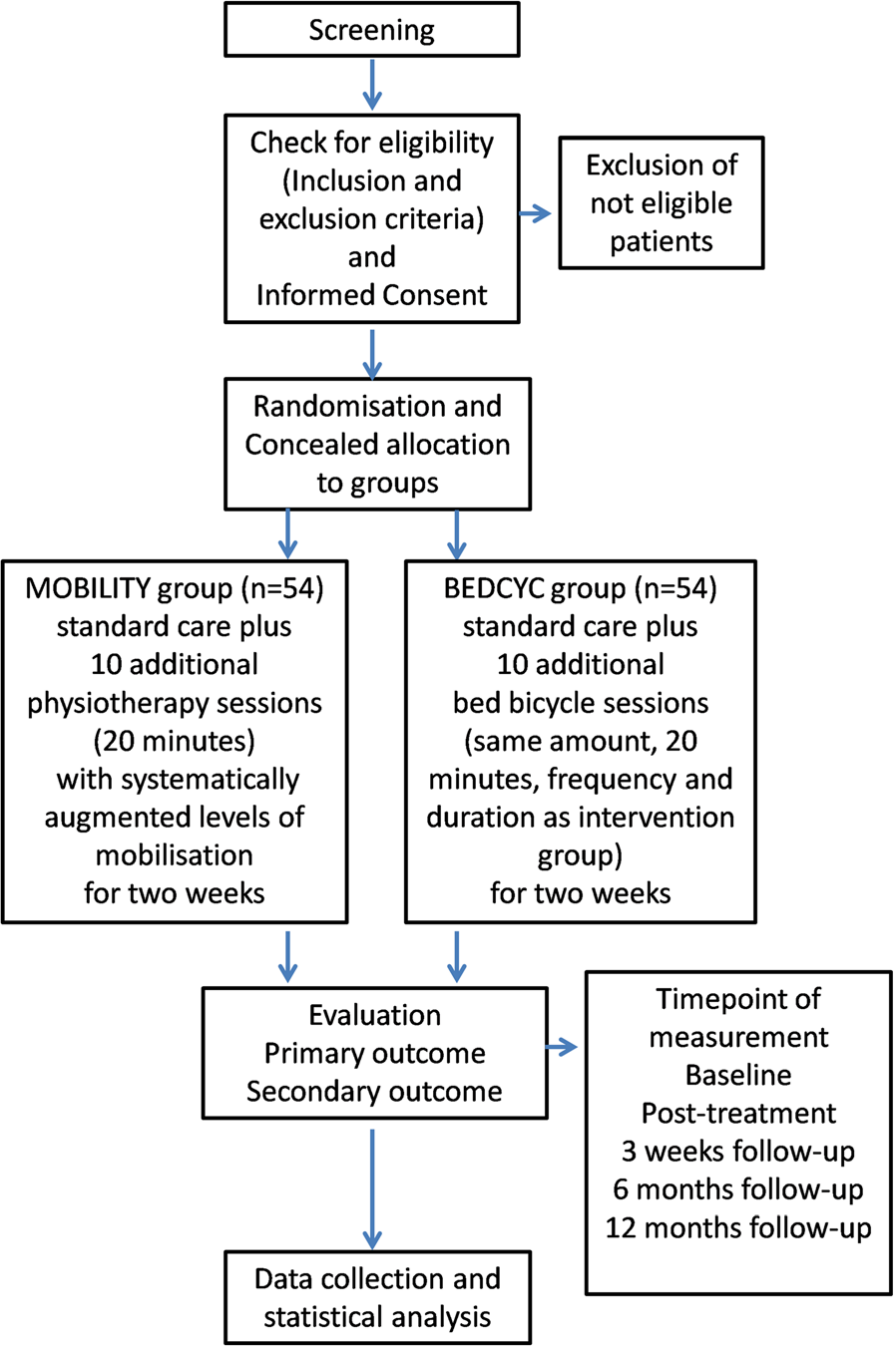
Standard Protocol Items: Recommendations for Interventional Trials (SPIRIT) figure for the schedule of enrolment, interventions and assessments. Time points: t0: baseline; t1: 2 weeks post treatment; t2: 3-week follow-up (FU1); t3: 6- and 12-month follow-ups (FU2); t4: 12-month follow-up (FU3). Abbreviations: FITonICU, Fitness and mobility training in patients with Intensive Care Unit-acquired muscle weakness; *FU* follow-up; *T* time point; *FAC* Functional Ambulation Categories; *FSS-ICU* Functional Status Score for the Intensive Care Unit Scored; *STS* ability to stand up from a chair independently; *MRC sum score* Medical Research Council (sum score of muscle strength of the upper (shoulder, elbow and wrist) and the lower limb (hip, knee and ankle)); *PFIT-S* Physical Function for the Intensive Care Unit Test-Scored; *EQ-5D* EuroQol 5 Dimensions questionnaire; *RNL-Index* Reintegration to Normal Living Index

This is a assessor-blinded, parallel-group, single-centre RCT of people with ICUAW with a defined diagnosis of CIM and/or CIP based on a protocol prepared according to the SPIRIT Statement (www.spirit-statement.org) [23] (see Additional file 1 and for the SPIRIT figure see Fig. 1).

The study will be conducted at the post-acute ICUs and the weaning units in one large specialised hospital (Klinik Bavaria Kreischa, Germany).

Prior to the intervention, patients will be randomised in a 1:1 ratio into one of two intervention parallel groups. The one experimental group will receive, in addition to standard ICU treatment, physiotherapy with systematically augmented levels of mobilisation (five times per week, over 2 weeks; each session will last 20 min; with a total of 10 additional sessions). The other group will receive in addition to standard ICU treatment in-bed bicycle sessions (with the same number, frequency and treatment time as the intervention group).

After receiving ethical approval and registration of the study we started recruiting in our hospital in 2016 and the final assessments including follow-up will be made in about 2020.

### Screening process

**Fig. 2:**
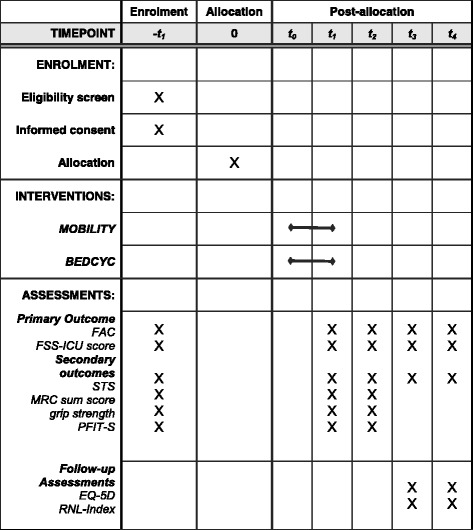
Flow chart of the Fitness and mobility training in patients with Intensive Care Unit-acquired muscle weakness (FITonICU) study design

We will enter all patients with the diagnosis of a defined CIP and/or CIM into our screening log. Subsequently, the patient will be screened for eligibility with the inclusion and exclusion criteria as outlined in Table 1 and Fig. 2. We will fully inform eligible patients and/or their caregivers about the study, and we will assign an identification number to the patient. Patients and/or their caregivers are then asked to provide informed consent. In the event that a potential participant does not meet the inclusion criteria, we will not retain identifiable information on that patient. We will report all reasons for exclusion in a flow chart. After providing informed consent we will record all medical and neurological information at baseline in a Case Report Form (Table 2).

**Table 1:** Inclusion and exclusion criteria of study

Inclusion criteria
1. Patient is in the post-acute phase and chronically critically ill, defined as more than 21 days ICU of treatment including mechanical ventilation, and at least 14 days more in an existing critical situation with the need for ICU treatment [27, 51, 52]
2. Acquired muscle weakness defined as a Medical Research Council (MRC) sum score of <48 points [1]
3. Defined diagnosis of critical illness myopathy (CIM) and/or polyneuropathy (CIP) confirmed by a neurologist according to published diagnostic criteria for CIP/CIM [24]
4. Older than 18 years of age
5. Richmond Agitation Sedation Scale (RASS) score from −1 to 1 [53]
6. Written informed consent of the patient or their legal guardian has been obtained
Exclusion criteria
1. Patients receiving palliative care
2. Comorbidities of the trunk or the lower limbs interfering with upright posture and walking function (e.g. amputation or fracture of lower limb)
3. Other neuromuscular or neurological disease and/or syndromes causing weakness in patients in the ICU (we will exclude patients with diseases and syndromes causing weakness in patients in the ICU [9], due to Guillain-Barré syndrome, myasthenia gravis, porphyria, Eaton-Lambert syndrome, amyotrophic lateral sclerosis, vasculitic neuropathy, cervical myelopathy and botulism)
4. Severe physical comorbidity before becoming critical ill (e.g. frailty due to neurological conditions)

**Table 2:** Overview of enrolment, interventions and assessments of the Fitness and mobility training in patients with Intensive Care Unit-acquired muscle weakness (FITonICU) study

Time point	Enrolment pretreatment	Allocation	Baseline (T0)	2 weeks post treatment (T1)	Daily documentation	3-week follow-up (FU1)	6- and 12-month follow-up (FU2–3)
Enrolment							
Eligibility screening	x						
Informed consent	x						
Allocation	x	x					
Interventions							
Physiotherapy			x	x	x	x	
Occupational			x	x	x	x	
therapy							
Other therapies (e.g. groups)			x	x	x	x	
Additional			x	x			
interventions			x	x			
Primary outcome							
FAC score			x	x	x	x	x
FSS-ICU			x	x		x	x
Secondary outcomes							
STS			x	x	x	x	x
MRC sum score			x	x		x	
Grip strength			x	x		x	
PFIT-S			x	x			
EQ-5D						x	x
RNL-Index						x	x

*FU* follow-up, *T* time point, *FAC* Functional Ambulation Categories, *FSS*-*ICU* Functional Status Score for the Intensive Care Unit Scored, *STS* ability to stand up from a chair independently, *MRC sum score* Medical Research Council (sum score of muscle strength of the upper (shoulder, elbow and wrist) and the lower limb (hip, knee and ankle)), *PFIT*-*S* Physical Function for the Intensive Care Unit Test-Scored, *EQ*-*5D* EuroQol 5 dimensions questionnaire, *RNL*-*Index* Reintegration to Normal Living Index

Two of the investigators will conduct all baseline assessments (see Table 2 for details). Those study assessors will not be involved in treating patients or with administration of the intervention and will remain fully blinded to the patient’s group allocation throughout the whole trial.

### Informed consent

Written informed consent for study participation will be obtained at 1 to 7 days after admission at our department.

Participants will be informed that successful completion of the baseline tests is required prior to randomisation to an intervention group.

### Eligibility criteria

Participants will have ICUAW due to defined CIP and/or CIM (for details see Table 1). The diagnosis of CIP and/or CIM will be confirmed by a neurologist according to published criteria [9, 24] at ICU prior to admission or after admission to our post-acute ICU.

### Outcome measures

Primary efficacy endpoints are walking ability and activities of daily living.

We define our endpoint walking ability with a category of ≥3 on the Functional Ambulation Categories (FAC; 0–5 score range) [25–27]. We use this scale to measure walking ability because in a recent cohort study it has been shown to be a very feasible tool to describe the ability to walk in different settings, such as ICU, post-acute rehabilitation and also at follow-up [26].

We will use the Functional Status Score for the Intensive Care Unit Scored (FSS-ICU; 0–35 score range) [28] to measure elementary activities of daily living. We will use this scale because in a recent cohort study the FSS-ICU was also very feasible, reliable and also predictive (for important aspects of daily living) in different settings and stages such as ICU, post-acute rehabilitation and post-acute care [26].

Our secondary endpoints comprise scales with a comprehensive spectrum of impairments, activities and participation for this population (see Table 2). We will measure the ability to stand up by documenting the ability to stand up from a standardised chair independently (STS) [27]. We will measure muscle strength of the upper (shoulder, elbow and wrist) and lower limb (hip, knee and ankle) using the Medical Research Council (MRC) sum score and calculate and provide a MRC sum score for the upper and also for the lower limbs [1, 27, 29]. We will measure the handgrip strength for both hands using a hand dynamometer (Jamar handgrip dynamometer; Sammons Preston Rolyan, Bolingbrook, IL, USA) [27, 30–32]. We will use the Physical Function for the Intensive Care Unit Test-Scored (PFIT-S) [33] to measure specific ICU impairments. We will assess quality of life using the EQ-5D [34] and measure participation with the Reintegration to Normal Living Index (RNL-Index) [27, 35, 36]. All of these measures are frequently used in research and/or clinical practice dealing with this patient group. Table 2 gives a detailed overview of the variables used at each time point of study and the data collection schedule.

### Assessment of safety and adverse events

At each assessment the following parameters will be systematically recorded: recurrent, fatal or nonfatal cardiovascular or cerebrovascular events; referral to an acute hospital; and death. After each intervention, the treating physiotherapist will record the presence of self-reported pain, fatigue, dizziness and number and nature of falls, and note any other adverse events. Additionally, oxygen saturation, mean arterial pressure, and heart rate will be documented for every therapy session.

### Standardisation of assessments

Blinded assessors have systematically undergone competency training to ensure standardisation of data collection methods. This included theoretical learning about the assessments, demonstration and practice on volunteers (patients with ICUAW in our post-acute ICU) under the supervision of the study’s clinical research coordinators. Successful completion of competency training was confirmed by a competency checklist.

### Data collection

Masked outcome data are collected by blinded assessors at baseline (T0), after 2 weeks of therapy (T1), after a 3-week follow-up (FU1), at 6-month follow-up (FU2) and at 12-month follow-up (FU3) (see Fig. 2 and Table 2). Outcome data will be double-entered into two data tables by masked researchers who have no knowledge of, or access to, identifiable participant information or treatment assignment. The two data entries are compared, corrections made and also tested for inter-rater reliability between the blinded assessors.

### Randomisation and concealment of allocation

Each study participant will be randomly assigned to one of two intervention groups, either the MOBILITY group or the BEDCYC group. Allocation will be concealed within an opaque sealed envelope (Fig. 2). The computer-generated random allocation sequence list will be carried out (JM) using a random-number generator (randomizer.org). Assignments will be enclosed in sequentially numbered, opaque, sealed envelopes and stored at the central study centre. The persons who assess eligibility, obtain informed consent and enrol patients in the trial (ST and KM) have no knowledge of group assignment. After recruitment, the appropriate numbered, opaque, sealed envelope will be opened and the randomisation information will be given to the patient and therapists (but not to the outcome assessors and not to the statistician performing the analysis).

### Interventions

Patients will be randomly assigned to either the MOBILITY or the BEDCYC (see Fig. 2 and Table 2).

Patients in the MOBILITY group will receive, in addition to standard treatment, 20 min physiotherapy daily. This additional physiotherapy programme includes systematically augmented levels of therapy and mobilisation (Table 3) five times per week, over 2 weeks. The MOBILITY programme contains 10 standardised exercises in hierarchical order (from low grade to higher grade):

**Table 3:** Overview of the schedule for increasing the intensity of every therapy session in the MOBILITY group

Session#	Repetitions	Increase^a^	Sets	Rest between sets (s)
1	As many as possible^b^	n.a.	2	120
2	As in previous session + increase	At least by 1	2	120
3	As in previous session + increase	At least by 1	2	120
4	As in previous session + increase	At least by 1	2	120
5	As in previous session + increase	At least by 1	3	90
6	As in previous session + increase	At least by 1	4	90
7	As in previous session + increase	At least by 1	4	90
8	As in previous session + increase	At least by 1	4	60
9	As in previous session + increase	At least by 1	4	60
10	As in previous session + increase	At least by 1	4	45

^a^Therapists chose from our list of 10 standardised exercises the most demanding exercise for every session to adjust for individual differences and physical state on the day of the session

^b^One repetition minimum and a maximum of 10 repetitions even if more repetitions would be possible; if more than 10 repetitions are possible the task (e.g. starting position) itself will be augmented; level 0 means the maximum of repetitions on the first day/first session

1. Turning from the one side to the other side when lying in the supine position and alternate supine bridging/buttock lift
2. Protracting hands and arms up and down the legs while sitting
3. Reach and grasp objects from a table while sitting
4. Knee-bending while standing near the bed with assistance
5. Sit-to-stand exercises as fast as possible
6. Balance exercises while standing without hand(rail) support
7. Reach and grasp objects from a table while standing
8. Stepping while standing
9. Walking on a 10-m indoor floor
10. Climbing stairs

In every therapy session the highest possible of the above-mentioned 10 standardised exercises will be selected. For example, a patient who is able to sit on their own will practice exercise #4, which is bending the knees while standing near the bed with assistance. If the workout of the selected exercise of the MOBILITY programme or the starting position of the exercise is not possible the preceding exercise will be selected by the therapist. For example, a patient who is able to sit on their own but fails to stand up and bend his knees while standing near the bed with assistance, exercise #3 instead of #4 will be selected and trained in therapy.

All 10 exercises will be trained by qualified and instructed therapists and intensity increased progressively according to Table 3. The therapists will document the trained exercises, and total duration and intensity for each single session. The therapy session will be paused/interrupted or terminated if the patient (using continuously monitoring):

- Has a heart rate below 60 or above 180 beats per min for longer than 3 min
- Has a persistent SpO_2_ < 88% when lying or sitting for longer than 3 min
- Has a systolic blood pressure below 100 mmHg or above 180 mmHg for longer than 3 min
- Has a diastolic blood pressure below 50 mmHg or above 110 mmHg for longer than 3 min

The adherence of the MOBILITY programme will be documented in daily logs and reported.

All single exercises in the MOBILITY programme are well-known and used routinely under different circumstances for the target patients in our department. Our additional MOBILITY programme with systematically augmented levels of mobilisation and therapy is, however, not described in the medical literature yet. For this reason we underwent a pilot training programme testing the feasibility of the MOBILITY programme in our department with all above-mentioned criteria. We found in 10 patients in this pilot training that the MOBILITY programme might be feasible without any adverse event.

### BEDCYC

The BEDCYC group will receive, in addition to standard treatment, 20 min of in-bed cycle ergometer sessions (the same number, frequency and treatment time as the MOBILITY group) [22]. We chose in-bed cycling as comparator to compare two true active and potentially effective interventions. For instance Kho et al. recently described the feasibility and safety of in-bed cycle ergometry as part of routine ICU practice [22]. They described 181 patients on ICU receiving a total of 541 cycling sessions and described a very low (0.2%) event rate and concluded that the use of in-bed cycling as part of routine physical therapy interventions in ICU patients is feasible and appears to be safe [22]. We will use the same in-bed cycle therapy approach as described earlier; however, contrary to the CYCLE pilot protocol of Kho et al. [37] we will use 20-min in-bed cycle ergometer sessions instead of 30-min sessions. BEDCYC will be provided by two experienced therapists who are only providing BEDCYC therapy but not the therapy to the experimental group.

Adherence to the BEDCYC therapy will be documented in daily logs and reported.

To control for cointerventions (standard therapy) during the intervention phase of the study (T0 to T1) therapists will document the total duration and content of both standard and additional therapy. Content will be described for each 5-min block.

### Criteria for discontinuing or modifying allocated intervention in both groups

Therapy sessions will not occur if any of the following conditions are present:

- Body temperature below 36 °C or above 38.5 °C
- Heart rate below 40 or above 130 beats per min
- Persistent SpO_2_ < 90% when lying or sitting
- Mean arterial pressure below 60 mmHg or above 110 mmHg
- Negative-pressure wound therapy
- Chest drainage systems
- Catecholamine medication (vasopressor/inotropes) within the past 2 h
- Active myocardial ischemia or any unstable arrhythmia
- Sedation or severe agitation (Richmond Agitation and Sedation Scale score <1 or >2)
- Uncontrolled pain
- Rehabilitation goal has been changed to palliative care
- Rehabilitation team perception that therapy is not appropriate despite absence of the above criteria
- Refusal of consent

### Additional information

The standard care/treatment includes sitting balance exercise, stretching, positioning, sit-to-stand training, transfer training to get out of bed, strengthening exercise (in and outside of bed), stepping and assistive standing exercises as described previously [38]. The standard treatment will be about 45 min daily of individual physical therapy and the duration of therapy will be kept equal in both intervention groups. Total time spent in rehabilitative therapy will be recorded by so-called usual care intervention logs to report the number of physiotherapy and occupational therapy sessions received in the first 2 weeks of the study (as part of the usual care treatment). Information about medication and treatment will be recorded at baseline. Length of stay in ICU and the time spent in post-acute ICU and in rehabilitation will be documented (in days).

As described above, physiotherapists will record in every session the presence of self-reported pain, fatigue, dizziness, number and nature of falls and note other adverse events. The adherence to standard care will be described in daily logs and reported.

### Sample size

Until now, no trials and, therefore, no specific effect size of the selected interventions for this specific population are present in the peer-reviewed literature. Our sample size calculation of 45 patients per study arm is based on a difference of 6 points in the primary endpoint of the FSS-ICU sum score between the parallel groups after 2 weeks of treatment. We assume an alpha level of 5%, a statistical power of 80% (beta = 20%) and a standard deviation (SD) of 10 using a two-sample *t* test for mean differences. Our assumption is based on the theory that the one experimental group achieves a mean of 22 points and the other group achieves a mean of 16 points in the FSS-ICU test assuming a SD of 10 in the FSS-ICU sum score and assuming that the rate of recovery of walking ability will be 0.6 in the one and 0.4 in the other group. We used the SAS (SAS Institute Inc., Cary, NC, USA) power and sample size programme for our sample size calculation. Because we consider a 20% dropout rate, a total of 108 patients (54 per study arm) would be enrolled to ensure 45 subjects in both intervention arms.

### Recruitment

Based on our sample size calculation, 108 patients will be recruited from our post-acute ICU over the time course of 3 to 4 years. In a first cohort study in our hospital we recruited 150 patients with ICUAW and a diagnosis of CIM/CIP in approximately 18 months [26].

The research staff will follow the recruitment process of a previous study [26] and will screen lists of patients on a daily basis to recruit continuously and will monitor recruitment. Recruitment statistics for every week will be discussed to improve the recruitment and retention procedure. Based on this the research team discusses updates to recruitment and retention strategies if necessary. Based on our experience from our previous study we believe that it seems to be reasonable to recruit the anticipated sample size in our trial within 3 to 4 years.

### Statistical methods

We will conduct both intention-to-treat and per-protocol analyses, with the intention-to-treat analysis being always the primary analysis for the primary and secondary outcomes.

Our descriptive statistics will include means and SDs, medians and interquartile ranges for continuous variables, and the number and proportions for categorical variables as appropriate [39].

We will compare the two intervention groups at baseline regarding characteristics and demographics, using two-tailed Student’s *t* tests or Fisher’s exact tests, as appropriate. Variables that differ between the groups at baseline will be considered as possible confounders and adjusted for in subsequent analyses. The global alpha level will be set at 0.05 for all comparisons. To avoid multiplicity we will use a Tukey-Kramer alpha adjustment for multiple comparisons of the same outcome. Skewed data will be analysed with nonparametric test alternatives (e.g. we will use the Mann-Whitney *U* test instead of Student’s *t* tests if data are skewed).

For the primary endpoint, the FSS-ICU, we will test the hypothesis that the sum score after 2 weeks of intervention and at follow-up will be statistically different between groups. We will consider a minimum difference of 5 points of the FSS-ICU sum score (range 0–35 points) between groups as being clinically important.

For the primary endpoint, walking ability, we will analyse the time to regain walking ability (defined as FAC score ≥3) in a time-to-event analysis. We will calculate the probability of regaining walking ability with the Kaplan-Meier (KM, ‘survival analysis’) method [40] and will provide KM plots as appropriate with 95% confidence intervals (CI) including the number of participants at risk at each time. We will report median times until regaining walking ability with 95% CI. The assumptions of KM analysis will be tested with the implemented function of SAS/STAT 9.3 (SAS Institute Inc., Cary, NC, USA). Cox regression analysis will be used to estimate relative hazard rates of the intervention groups [41]. Time to event will be defined as the time difference between study entry (T0) and the date of reaching a FAC score equal to 3, or the possible censoring dates of discharge or treatment refusal or death, respectively. Data will, therefore, be censored appropriately if patients are discharged, refused treatment or died. We will use the log-rank test to test statistically for differences between the KM curves of the intervention groups [42]. We will use a stepwise multivariable Cox regression analysis with a variable selection to also take into account the influence of all dependent variables (PROC PHREG; best subset analysis; SAS 9.3, SAS Institute Inc., Cary, NC, USA) [41, 43]. The following dependent-variable categories will be analysed for their association with walking ability:

- Demographic variables at baseline (such as age and sex)
- All clinical variables at baseline (such as muscle strength, PFIT-S; Table 2) and
- Medical characteristics at baseline (such as diagnosis and duration of primary illness)

All dependent (explanatory) variables will be first described in a univariate analysis and then selected for a multivariable model based on statistical significance [44–46]. In this process an explanatory variable has to be significant at the 0.2 level to be *entered* into the multivariate model [43]. To *remain* in the multivariate model an explanatory variable has to be significant at the 0.1 level [43]. We will first select explanatory variables from a list of all univariate analysis with the highest global chi-square score into our multivariable model [43]. We will use a graphical inspection and also evaluate whether the proportional hazard assumption for an explanatory variable is met [41]. We will, for the final model selection, compare the multivariate models (with remaining variables) on the global score chi-square statistic (best subset selection) and on the Akaike’s information criterion (AIC) performed [43]. The aim of our analysis is to *explain* the dependent variable (e.g. regaining walking function) by a multivariate Cox proportional hazard model with not too many variables (to prevent overfitting) [43]. The multivariate effects of explanatory variables in the final model will be expressed as hazard ratios (HR) with 95% CI [43]. We will use SAS/STAT 9.3 for all statistical procedures (SAS Institute Inc., Cary, NC, USA).

We will do an interim analysis for efficacy and ineffectiveness after half the patients have completed our study trial. The trial will be stopped if there is a statistically significant difference between the groups in one direction at an alpha level of 0.05 at this time point of recruitment.

### Adverse event monitoring and reporting

All adverse events will be carefully monitored at every level of the FITonICU study. A Data Safety Monitoring Board provides oversight and meets after randomisation of every 20 newly recruited patients. All adverse events are reported immediately to the responsible physician and the board members are informed of all adverse events.

The Data Safety Monitoring Board and the research team (all authors) will be responsible for data safety and for the confidentially of recruited patients. The confidentiality of participants will be granted by using anonymous ID lists for all recruited patients. All patient data will be maintained and stored confidentially on a separate computer server.

The Data Safety Monitoring Board will do an interim analysis for safety of every 20 included and treated patients. The trial will be stopped:

- If there is a statistically significant difference in safety between the groups in one direction at an alpha level of 0.05 at this time point of recruitment
- If one of the interventions is associated with unexpected excessive adverse effects such as skin ulcerations, pulmonary embolism or deep vein thrombosis, or there are excessive withdrawals
- If the study recruitment is unsuccessful (e.g. 3 months without any recruitment) or
- If other situations occur that might justify stopping this trial

### Ethics and dissemination

The FITonICU study will be conducted in accordance with the Helsinki Declaration. The study is noninvasive, imposes no additional risk on patients, seems to have enough power to detect meaningful determinants and our protocol has been approved by the medical ethical committees (ethical vote ‘Landesärztekammer Sachsen’ Germany; EK-BR-104/15-1). Furthermore, written informed consent is obtained from all participants or, if necessary, from a legal guardian (WS, UB and ST). We registered our study on 6 April 2016 before enrolling the first patient in the trial at the German Clinical Trials Register (www.germanctr.de/) with the identifier DRKS00010269.

We plan to disseminate the results of this study to the scientific, medical and general public by publication in national and international peer-reviewed journals, as well as by presentations at conferences and meetings with clinicians working with patients with ICUAW.

Authorship will follow the recommendations of the International Committee of Journal Editors for authorship. No professional writers will be employed.

## Discussion

The FITonICU study will be one of the first rater-blinded RCTs comparing the effects of two active physical rehabilitation interventions for people with ICUAW and defined CIP and/or CIM to improve walking and other activities of daily living. The results of this trial might help to close an obvious research gap for physical rehabilitation of people with ICUAW [21].

Considering the lack of evidence-based rehabilitation, and the high level of medical interest in the area of rehabilitation of people who are chronically critically ill, this trial will be a significant advance in physical rehabilitation for people with ICUAW due to a defined CIP and/or CIM.

Although descriptions of post-acute interventions and long-term studies for people who are chronically critically ill do exist [18, 47–49], there are no RCTs that measure the effects of potentially effective physical rehabilitation interventions for people who are critically ill with ICUAW on improvement in activities and physical function [21]. This study will, therefore, determine whether a specific physical intervention for people with ICUAW is beneficial in the post-acute care of these patients.

### Limitations

One could argue that the lack of a true control group, e.g. of standard or no additional physiotherapy, might be a weakness of our trial protocol. We have, however, designed this study to compare two potentially capable and reasonable active physical rehabilitation interventions for patients on ICU. The feasibility and safety of BEDCYC was established in previous studies [37] and is a promising physical rehabilitation intervention for patients on ICU. The efficacy and effectiveness relative to standard treatment alone for BEDCYC will be investigated in other RCTs [37, 50]. The MOBILITY intervention is not yet described in the literature, but MOBILITY was tested over several months at our department and seems a feasible physical rehabilitation intervention for patients on ICU. We decided not to introduce a ‘classical’ control group in our study, because there is no such classical control treatment in the ICU, but there is a need for effective therapy to be conducted to explore the role of physical rehabilitation interventions for people with CIP and CIM.

The second limitation is that this trial is planned to be carried out as a single-centre study and can, therefore, only be seen as a first or pilot study. This pilot trial is, however, adequately powered and funded. Further RCTs should, however, try to recruit further centres and hospitals to increase the clinical knowledge about physical rehabilitation interventions for people with ICUAW.

### Trial status

Patient recruitment began on 15 April 2016 and is expected to continue for 3 to 4 years in total.

## Additional file

Additional file 1: SPIRIT 2013 Checklist: Recommended items to address in a clinical trial protocol and related documents*. (PDF 43 kb)

## Abbreviations

AIC: Akaike’s information criterion
CI: Confidence intervals
CIM: Critical illness myopathy
CIP: Critical illness polyneuropathy
EQ-5D: EuroQol 5 dimensions questionnaire
FAC: Functional Ambulation Categories
FITonICU: Fitness and mobility training in patients with Intensive Care Unit-acquired muscle weakness
FSS-ICU: Functional Status Score for the Intensive Care Unit-Scored
FU: Follow-up
ICU: Intensive care unit
ICUAW: Intensive-care-unit-acquired muscle weakness
KM: Kaplan and Meier
MRC: Medical Research Council
PFIT-S: Physical Function for the Intensive Care Unit Test-Scored
RNL-Index: Reintegration to Normal Living Index
STS: Ability to stand up from a chair independently (sit-to-stand)
T: Time point
